# The association between phenotypes of polycystic ovary syndrome and metabolic dysfunction-associated fatty liver disease

**DOI:** 10.3389/fendo.2025.1480528

**Published:** 2025-08-04

**Authors:** So-hyeon Hong, Yeon-Ah Sung, Young Sun Hong, Do Kyeong Song, Hyein Jung, Kyungah Jeong, Hyewon Chung, Hyejin Lee

**Affiliations:** ^1^ Division of Endocrinology and Metabolism, Department of Internal Medicine, Ewha Womans University College of Medicine, Seoul, Republic of Korea; ^2^ Department of Obstetrics and Gynecology, Ewha Womans University College of Medicine, Seoul, Republic of Korea

**Keywords:** polycystic ovary syndrome, metabolic-associated fatty liver disease, hyperandrogenism, oligomenorrhea, polycystic ovary morphology

## Abstract

**Introduction:**

Polycystic ovary syndrome (PCOS) is associated with an increased risk of non-alcoholic fatty liver disease (NAFLD). With the introduction of the new definition of metabolic dysfunction-associated fatty liver disease (MAFLD), there has been a lack of studies investigating the prevalence and clinical characteristics of PCOS and its phenotypes, including hyperandrogenism (HA), oligoanovulation (OA), and polycystic ovarian morphology (PCO) in association with MAFLD. The aim of this study is to explore MAFLD prevalence in young women with PCOS and determine the independent impact of PCOS phenotypes on MAFLD.

**Methods:**

This cross-sectional study included 1,422 women with PCOS diagnosed using the Rotterdam criteria, the presence of at least two of three diagnostic criteria: 1) hyperandrogenism (HA), 2) oligoanovulation (OA), and 3) polycystic ovary morphology (PCO).

**Results:**

Among women with PCOS, 31.2% had NAFLD, and 65.1% of them were diagnosed with MAFLD. In PCOS phenotypes, MAFLD prevalence was 25.1% for HA+OA+PCO, 27.6% for HA+OA, 8.8% for HA+PCO, and 13.0% for OA+PCO. Women with PCOS and HA+OA+PCO had higher odds of MAFLD (OR [95% CI] of 1.47 [1.04–2.09]), as did those with HA+OA (1.87 [1.18–2.96]), after adjusting for demographic and clinical factors. However, the association between women with PCOS and HA+PCO and MAFLD was not statistically significant (0.51 [0.21–1.24]).

**Discussion:**

In women with PCOS, both HA+OA+PCO and HA+OA phenotypes were independently associated with MAFLD. HA and OA may contribute independently to the higher prevalence of MAFLD in these individuals.

## 1 Introduction

Polycystic ovary syndrome (PCOS) is a prevalent endocrine disorder among women. It includes medical conditions such as hyperandrogenism (HA), oligoanovulation (OA), and polycystic ovary morphology (PCO) (1). Younger women with PCOS usually experience reproductive problems such as menstrual irregularity and infertility. However, in the longer run, they have an increased risk of developing metabolic diseases, including obesity, diabetes mellitus, dyslipidemia, and non-alcoholic fatty liver disease (NAFLD) (2).

NAFLD is defined as hepatic steatosis occurring in the absence of alcohol consumption or other known causes of liver disease (3). However, the term NAFLD could be limited in its application and does not incorporate metabolic comorbidities and insulin resistance associated with hepatic fat accumulation. Therefore, in 2020, an international consensus of liver experts proposed a change in terminology from NAFLD to metabolic dysfunction-associated fatty liver disease (MAFLD) (4). The new MAFLD nomenclature offers superior predictive ability for identifying patients at high risk of hepatic disease progression and cardiovascular diseases (CVD) (5). Furthermore, recent studies have revealed differences in CVD among the MAFLD subtypes (6).

Previous studies have demonstrated that women with PCOS have increased NAFLD risk (7, 8). Insulin resistance and obesity underly the pathology of both PCOS and NAFLD. These conditions can exacerbate hepatic *de-novo* lipogenesis, rendering the liver susceptible to injury from oxidative stress, and ultimately leading to liver inflammation and fibrosis (9). However, no study has examined the prevalence of MAFLD and its subtypes in PCOS. Furthermore, data from studies investigating the impact of PCOS diagnostic components (HA, OA, PCO) on NAFLD development have yielded inconsistent results. To date, no study has examined the relationship between PCOS diagnostic components and MAFLD.

This study aimed had two primary objectives: first, to explore the prevalence of MAFLD and its subtypes among young women with PCOS, and second, to examine the independent influence of PCOS phenotypes and associated diagnostic components on MAFLD.

## 2 Materials and methods

### 2.1 Study design and population

This was a cross-sectional study of women with PCOS, aged 18 years or older and of reproductive age, who visited the endocrinology and gynecology clinic at Ewha Womans University Mokdong Hospital between December 2008 and December 2010. PCOS was diagnosed if two of the following Rotterdam criteria were present: i) HA, ii) OA, or iii) PCO (10). HA was defined as biochemical hyperandrogenemia based on total testosterone ≥ 67 ng/dL or free testosterone ≥ 0.84 ng/dL (total testosterone thresholds were calculated based on testosterone level exceeding the 95^th^ percentile in a reference group of 1,120 healthy women with regular menstruation cycle) (11). OA was defined as oligomenorrhea, characterized by a menstrual cycle length > 35 days or fewer than 8 menstrual periods per year, or as amenorrhea. Patients with other androgen excess disorders, such as congenital adrenal hyperplasia (specifically non-classical congenital adrenal hyperplasia due to 21-hydroxylase deficiency, defined as a 17-hydroxyprogesterone level > 2 ng/mL), Cushing’s syndrome (a cortisol level > 1.8 μg/dL following the 1 mg overnight dexamethasone suppression test), hyperprolactinemia, or androgen-producing neoplasms were excluded. Patients who had taken oral contraceptives or metformin for 3 months prior to enrolling in the study, and were heavy users of alcohol were excluded. We obtained written informed consent from all participants, and conducted according to the Declaration of Helsinki. The study received approval from the Institutional Review Board of Ewha Women’s University Mokdong Hospital (IRB No. 187-30).

### 2.2 Anthropometric and biochemical measurements

All participants visited the clinic on the 3^rd^ day of their menstrual cycle following an overnight fast of at least 8 hours. Participant height and weight were measured over light clothing. Body mass index (BMI) was calculated as body weight (kg) divided by height squared (m^2^). Blood pressure was measured with the participant in the seated position and calculated as the mean of two manual sphygmomanometer readings.

Venous blood samples were collected. Plasma glucose levels were determined using a glucose oxidase method (Beckman Model Glucose Analyzer 2, CA, USA). Serum aspartate transaminase (AST), alanine aminotransferase (ALT), gamma-glutamyl transferase (GGT), triglycerides, total cholesterol, and high-density lipoprotein (HDL) cholesterol were quantified using an enzymatic assay conducted with an automated analyzer (Hitachi 7150 Automatic Chemistry Analyzer, Tokyo, Japan). Total testosterone levels were measured using a chemiluminescent immunoassay (Siemens, NY, USA). Sex hormone-binding globulin (SHBG) levels were measured using an immunoradiometric assay (DPC, Los Angeles, CA, USA). Free testosterone levels were determined by calculating them based on the measurements of total testosterone, SHBG, and albumin levels, using a formula established by the International Society for the Study of the Aging Male (12).

### 2.3 Biomarkers of NAFLD and MAFLD

NAFLD was determined using a hepatic steatosis index (HSI) using anthropometric and biochemical measurements. NAFLD was calculated using the following formula: 8 × AST/ALT + BMI (+2 if diabetes mellitus, +2 if female) (13). A HSI index > 36 was indicative of NAFLD. Several studies have validated the diagnostic performance of HSI in determining NAFLD (14–16).

MAFLD was defined as the presence of hepatic steatosis by HSI, and one or more of the below conditions:1) overweight or obesity (BMI ≥ 23 kg/m according to the World Health Organization Asia-Pacific Criteria); 2) type 2 diabetes mellitus; 3) presence of at least two of the following metabolic abnormalities—waist circumference ≥ 88 cm, hypertension ≥ 130/85 mmHg, dyslipidemia (TG ≥ 150 mg/dL or HDL cholesterol < 50 mg/dL), prediabetes (fasting glucose 100–125 mg/dL or Hemoglobin A1C 5.7%–6.4%), C-reactive protein > 2 mg/L, or insulin resistance (HOMA-IR ≥ 2.5) (4). Based on the metabolic profile, MAFLD was categorized into the following four distinct subtypes: 1) MAFLD coexisting with type 2 diabetes mellitus (DM-MAFLD), 2) MAFLD associated with overweight or obesity, along with metabolic abnormalities (OW-MAFLD with MA), 3) MAFLD linked to overweight or obesity but devoid of metabolic abnormalities (OW-MAFLD without MA), 4) Lean-MAFLD.

### 2.4 Statistical analyses

Baseline characteristics are reported as mean ± standard deviation for continuous variables and numbers (%) for categorical variables. The Student’s *t*-test or Wilcoxon rank-sum test was used to compare the two groups depending on the normality of distribution of variables. Comparisons between the three groups were conducted using analysis of variance (ANOVA) or the Kruskal-Wallis test. Categorical variables were analyzed by the χ^2^ test or Fisher’s exact test. To examine the associations between PCOS and its phenotypes with MAFLD and its subtypes, we conducted multiple logistic regression analyses. The results were presented as odds ratios (OR) and corresponding 95% confidence interval (CI). Analyses were conducted in models adjusted for possible confounders. Model 1 was adjusted for age, and model 2 was adjusted for age, systolic blood pressure (SBP), diastolic blood pressure (DBP), as well as levels of AST, ALT, GGT, triglycerides, total cholesterol, and fasting glucose. A *P* value < 0.05 was considered statistically significant for all analyses. All statistical analyses were performed using SPSS version 20.0 (IBM Corp., Chicago, IL, USA).

## 3 Results

### 3.1 Baseline characteristics

A total of 1,422 women with PCOS were identified. After excluding participants with chronic liver disease (n = 6) and those with incomplete information (n = 20), 1, 396 participants were included in the study cohort. NAFLD prevalence was 31.2%, as assessed using the HSI. Out of the 435 participants with NAFLD, 283 (65.1%) participants were diagnosed with MAFLD. Of whom, 5 (1.8%) participants had DM-MAFLD, 56 (19.8%) participants had OW-MAFLD with MA, and 222 (78.4%) participants had were OW-MAFLD without MA. None of the participants had Lean-MAFLD (**Supplementary Figure 1**).

**Figure 1:**
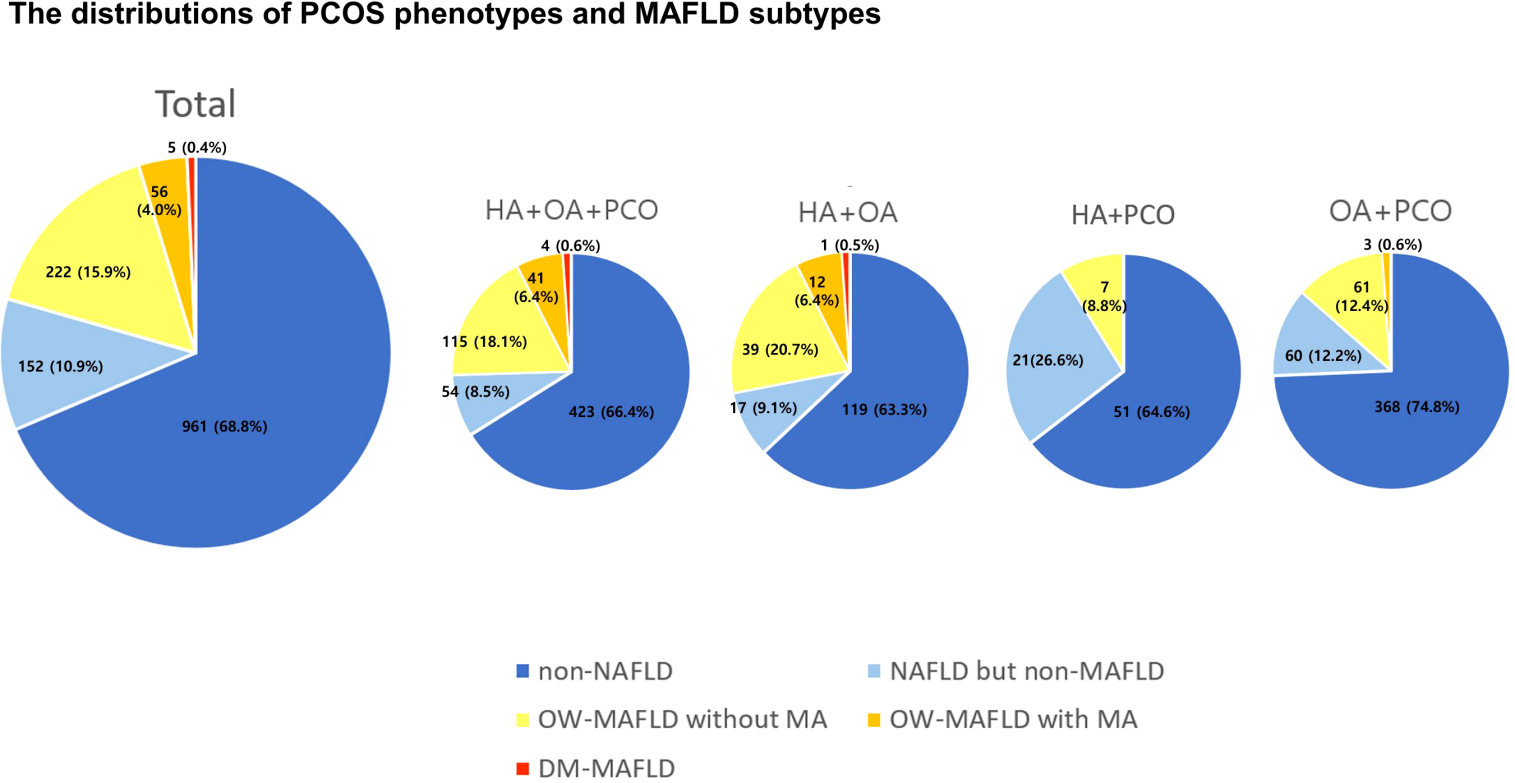
The distributions of PCOS phenotypes and MAFLD subtypes. PCOS, polycystic ovary syndrome; MAFLD, metabolic dysfunction-associated fatty liver disease; HA, hyperandrogenism; OA, oligoanovulation; PCO, polycystic ovary morphology; OW, overweight; MA, metabolic abnormalities.

The prevalence and distribution of MAFLD subtypes in PCOS phenotypes are presented in **Figure 1**. Across all four PCOS subtypes, non-NAFLD was the most prevalent, women with OA+PCO comprised the largest proportion (74.8%) of the cohort. In PCOS phenotypes, 25% of women with HA+OA+PCO had MAFLD, 25% of women with HA+OA had MAFLD, 14% of women with OA+PCO had MAFLD, and 9% of women with OA+PCO had MAFLD. In PCOS women with HA+OA+PCO, the second most prevalent subtype was OW-MAFLD without MA (18.1%), succeeded by NAFLD (8.5%), OW-MAFLD with MA (6.4%), and DM-MAFLD (0.6%). Women with PCOS and HA+OA exhibited similar trends. In PCOS women with HA+PCO, NAFLD but non-MAFLD was the second most common subtype, followed by OW-MAFLD without MA, whereas OW-MAFLD with MA and DM-MAFLD were absent. In PCOS women with OA+PCO, the prevalence of NAFLD but non-MAFLD and OW-MAFLD without MA was similar, followed by OW-MAFLD with MA.

The baseline characteristics of the study participants based on the presence or absence of NAFLD are shown in **Table 1**. Women with NAFLD were more likely to be obese, have higher systolic and diastolic blood pressure, higher levels of AST, ALT, GGT, total cholesterol, triglyceride, and fasting glucose, and exhibited lower HDL cholesterol levels compared with women without NAFLD. Women with NAFLD had significantly higher levels of free testosterone than those without NAFLD, but there were no significant differences in the levels for total testosterone. The prevalence of HA was significantly higher in women with NAFLD than in those without NAFLD. The prevalence of OA and PCO did not exhibit statistically significant differences between women with and those without NAFLD.

**Table 1:** Baseline characteristics of women with PCOS according to presence of NAFLD.

Variable	Non-NAFLD	NAFLD	*P* value
n (%)	961 (68.8)	435 (31.2)	
Age (years)	25 ± 5	24 ± 5	0.95
BMI	20.7 ± 2.4	25.6 ± 4.9	<0.01
WC	72.3 ± 7.1	82.5 ± 11.9	<0.01
SBP	108 ± 11	113 ± 12	<0.01
DBP	68 ± 9	73 ± 9	<0.01
AST	20 ± 8	25 ± 16	<0.01
ALT	20 ± 10	20 ± 18	0.77
GGT	13 ± 8	17 ± 13	<0.01
Total cholesterol	179 ± 31	182 ± 29	0.048
Triglyceride	80 ± 43	100 ± 61	<0.01
HDL cholesterol	56 ± 14	48 ± 11	<0.01
Fasting glucose	85 ± 9	88 ± 12	<0.01
Total testosterone	67.7 ± 19.3	68.5 ± 19.7	0.48
Free testosterone	0.77 ± 0.4	0.98 ± 0.50	<0.01
Hyperandrogenism (%)	593 (61.7)	311 (71.5)	<0.01
Oligoanovulation (%)	910 (94.7)	407 (93.6)	0.39
Polycystic ovary morphology (%)	842 (87.6)	366 (84.1)	0.09

Values are presented as mean ± standard deviation or n (%).

PCOS, polycystic ovary syndrome; NAFLD, nonalcoholic fatty liver disease; BMI, body mass index; WC, waist circumference; SBP, systolic blood pressure; DBP, diastolic blood pressure; AST, aspartate transaminase; ALT, alanine aminotransferase; GGT, gamma-glutamyl transferase; HDL, high-density lipoprotein.

Among the 435 women with PCOS and NAFLD, 283 (65.1%) were diagnosed with MAFLD (**Table 2**). Women with MAFD were more obese and presented with more unfavorable metabolic parameters than their counterparts who had NAFLD without MAFLD. Women with PCOS and MAFLD had higher levels of free testosterone, and a higher prevalence of HA and OA than in those without MAFLD. PCO prevalence was not significantly different in either group. According to MAFLD subtypes, BMI and the levels of AST, ALT, GGT, total cholesterol, triglyceride, and fasting glucose were highest in the DM MAFLD group, followed by the OW-MAFLD with MA, and OW-MAFLD without MA groups. Similar trends were observed in the levels of total testosterone and free testosterone, as well as in the prevalence of HA.

**Table 2:** Baseline characteristics of women with NAFLD and PCOS stratified by MAFLD and its subtypes.

Variable	Non- MAFLD	MAFLD	*P*	MAFLD			*P*
				OW- MAFLD without MA	OW-MAFLD With MA	DM-MAFLD	
n (%)	152 (34.9)	283 (65.1)					
n (% in MAFLD)				222 (78.4)	56 (19.8)	5 (1.8)	
Age (years)	23 ± 4	25 ± 5	<0.01	25 ± 6	27 ± 6	26 ± 8	0.07
BMI	20.7 ± 1.4	28.2 ± 4.0	<0.01	27.3 ± 3.6	31 ± 5.3	34 ± 5.3	<0.01
WC	71.4 ± 5.0	88.5 ± 10.1	<0.01	86.1 ± 9.1	96.9 ± 8.0	104 ± 12.1	<0.01
SBP	107 ± 9	116 ± 12	<0.01	112 ± 9	132 ± 10	127 ± 8	<0.01
DBP	70 ± 7	75 ± 10	<0.01	71 ± 7	88 ± 8	83 ± 4	<0.01
AST	20 ± 5	27 ± 18	<0.01	23 ± 10	42 ± 31	45 ± 20	<0.01
ALT	10 ± 3	25 ± 21	<0.01	20 ± 13	41 ± 31	57 ± 25	<0.01
GGT	11 ± 3	20 ± 15	<0.01	17 ± 11	29 ± 19	56 ± 33	<0.01
Total cholesterol	173 ± 25	188 ± 30	<0.01	185 ± 28	197 ± 31	188 ± 64	0.04
Triglyceride	71 ± 28	117 ± 68	<0.01	108 ± 61	148 ± 82	161 ± 44	<0.01
HDL cholesterol	53 ± 12	45 ± 10	<0.01	46 ± 11	43 ± 9	41 ± 8	0.07
Fasting glucose	83 ± 8	91 ± 12	<0.01	89 ± 9	93 ± 9	152 ± 22	<0.01
Total testosterone	67.0 ± 18.7	69.4 ± 20.2	0.22	67.7 ± 19.4	74.7 ± 21.2	81.2 ± 31.7	0.03
Free testosterone	0.68 ± 0.31	1.14 ± 0.51	<0.01	1.08 ± 0.50	1.30 ± 0.50	1.62 ± 0.37	<0.01
hyperandrogenism (%)	593 (61.7)	311 (71.5)	<0.01	161 (72.5)	53 (94.6)	5 (100)	<0.01
Oligoanovulation (%)	131 (86.2)	276 (97.5)	<0.01	215 (96.8)	56 (100)	5 (100)	0.43
Polycystic ovary morphology (%)	135 (88.8)	231 (81.6)	0.055	183 (82.4)	44 (78.6)	4 (80)	0.71

Values are presented as mean ± standard deviation or n (%).

NAFLD, nonalcoholic fatty liver disease; PCOS, polycystic ovary syndrome; MAFLD, metabolic dysfunction-associated fatty liver disease; OW, overweight; MA, metabolic abnormality; DM, diabetes mellitus; BMI, body mass index; WC, waist circumference; SBP, systolic blood pressure; DBP, diastolic blood pressure; AST, aspartate transaminase; ALT, alanine aminotransferase; GGT, gamma-glutamyl transferase; HDL, high-density lipoprotein.

### 3.2 The phenotype of PCOS and the risk of MAFLD

The association between PCOS phenotypes and the presence of MAFLD and its subtypes is presented in **Table 3**. In univariate analysis, the association between PCOS with HA+OA+PCO and MAFLD was OR (95% CI) of 2.24 (1.63–3.08), PCOS with HA+OA and MAFLD was 2.56 (1.69–3.87), and PCOS with OA+PCO and MAFLD was 0.65 (0.29–1.48). These results remained statistically significant even after adjusting for multiple variables including age, SBP, DBP, AST, ALT, GGT, triglyceride, total cholesterol, and fasting glucose, (OR (95% CI) of 1.47 (1.04–2.09) for HA+OA+PCO, 1.87 (1.18–2.96) for HA+OA, and 0.51(0.21–0.24) for HA+PCO). In contrast, women with PCOS and HA + PCO showed no statistically significant difference in the risk for MAFLD compared with women with PCOS and OA + PCO. In the case of OW-MAFLD without MA, women with HA+OA+PCO and HA+OA phenotypes had increased odds compared with PCOS women with OA+PCO in univariate analysis. However, after adjusting for multiple variables, the association remained significant only in HA+OA phenotype.

**Table 3:** Logistic regression analysis to determine the effect of MAFLD and its subtypes on PCOS phenotypes.

Variable	PCOS phenotypes			
	OA+PCO	HA+OA+PCO	HA+OA	HA+PCO
MAFLD				
Crude	Reference	2.24 (1.63-3.08) ¶	2.56 (1.69-3.87) ¶	0.65 (0.29-1.48)
Model 1	Reference	2.24 (1.63-3.09) ¶	2.75 (1.81-4.19) ¶	0.63 (0.28-1.44)
Model 2	Reference	1.47 (1.04-2.09) ¶	1.87 (1.18-2.96) ¶	0.51 (0.21-1.24)
OW-MAFLD without MA				
Crude	Reference	1.56 (1.11-2.18) ¶	1.85 (1.19-2.89) ¶	0.69 (0.30-1.56)
Model 1	Reference	1.55 (1.11-2.17) ¶	1.91 (1.22-2.98) ¶	0.68 (0.30-1.54)
Model 2	Reference	1.27 (0.89-1.81)	1.71 (1.08-2.71) ¶	0.60 (0.26-1.38)

Model 1: Adjusted for age.

Model 2: Adjusted for age, systolic blood pressure, diastolic blood pressure, aspartate transaminase, alanine aminotransferase, gamma-glutamyl transferase, triglyceride, total cholesterol, and fasting glucose.

¶: statistically significant.

MAFLD, metabolic dysfunction-associated fatty liver disease; PCOS, polycystic ovary syndrome; OA, oligoanovulation; PCO, polycystic ovary morphology; HA, hyperandrogenism; OW, overweight; MA, metabolic abnormalities.

We analyzed the influence of each PCOS diagnostic component on the risk of MAFLD (**Table 4**). After adjusting for multiple variables, HA was associated with a 1.47-fold (95% CI 1.04–2.09) increase in odds, whereas OA displayed a 2.86-fold (95% CI 1.21–6.79) increase in odds of developing MAFLD.

**Table 4:** Logistic regression analysis to determine the effects of MAFLD on PCOS components.

	PCOS components		
	HA	OA	PCO
MAFLD			
Crude	2.24 (1.63-3.08) ¶	3.45 (1.56-7.65) ¶	0.88 (0.61-1.27)
Model 1	2.24 (1.63-3.09) ¶	3.54 (1.59-7.86) ¶	0.81 (0.56-1.18)
Model 2	1.47 (1.04-2.09) ¶	2.86 (1.21-6.79) ¶	0.79 (0.52-1.19)
OW-MAFLD without MA			
Crude	1.56 (1.11-2.18) ¶	2.27 (1.02-5.05) ¶	0.84 (0.56-1.26)
Model 1	1.56 (1.11-2.19) ¶	2.29 (1.03-5.11) ¶	0.81 (0.54-1.22)
Model 2	1.56 (1.11-2.20) ¶	2.12 (0.94-4.83)	0.74 (0.48-1.12)

Model 1: Adjusted for age.

Model 2: Adjusted for age, systolic blood pressure, diastolic blood pressure, aspartate transaminase, alanine aminotransferase, gamma-glutamyl transferase, triglyceride, total cholesterol, and fasting glucose.

¶: statistically significant.

MAFLD, metabolic dysfunction-associated fatty liver disease; PCOS, polycystic ovary syndrome; OA, oligoanovulation; PCO, polycystic ovary morphology; HA, hyperandrogenism; OW, overweight; MA, metabolic abnormalities.

## 4 Discussion

In this study, young Korean women with PCOS exhibited a 31.2% prevalence of NAFLD as assessed using the HSI, and 65.1% of the women with PCOS had MAFLD. According to the MAFLD criteria, OW-MAFLD, particularly OW-MAFLD without MA was the most common MAFLD subtype. In PCOS phenotypes, approximately 25% of PCOS women with HA+OA+PCO or women with HA+OA had MAFLD. In PCOS women with OA+PCO, approximately 14% had MAFLD, and in the cohort of PCOS women with HA+PCO, approximately 9% had MAFLD. Women with PCOS and MAFLD had higher free testosterone levels and a higher prevalence of HA and OA than women with PCOS without MAFLD. Women with the HA+OA+PCO and HA+OA phenotypes showed a significant association with MAFLD compared to PCOS women with OA+PCO phenotype. Among PCOS diagnostic components, HA and OA were independently associated with MAFLD, whereas PCO was not associated with MAFLD.

To the best of our knowledge, this study is the first to examine the association between PCOS and MAFLD in young women. The incidence of MAFLD within the NAFLD population varies depending on age, ethnicity, and the presence of metabolic conditions. A study conducted in the US used the NHANES III score and showed that NAFLD prevalence was 16.5%, whereas MAFLD prevalence was 18.1% (17). In that study, 49.5% of the cohort comprised males, with an average age of 43 years. Additionally, 15.4% of the patients with MAFLD were identified as heavy alcohol users. Another study, which employed the Korean National health screening database, examined a cohort comprising 49% males with an average age of 50 years and observed an MAFLD prevalence of 36.7% (6). In a meta-analysis of approximately 380,000 individuals, the prevalence of MAFLD was 39.22% and that of NAFLD was 33.86%. Among individuals with MAFLD, 82% were also diagnosed with NAFLD (18). Our study cohort included young females with PCOS who were not heavy alcohol users. This could explain the lower MAFLD prevalence observed in this cohort. Additionally, the low incidence of metabolic abnormalities among these women could have impacted the incidence rate of MAFLD. Therefore, although PCOS is a risk factor for NAFLD and MAFLD, its prevalence in this study appears to be relatively low compared with that in other studies.

Interestingly, among the 283 women with PCOS, cases of lean-MAFLD were absent and approximately 98% had OW-MAFLD, with the majority under the category of OW-MAFLD without MA. Lee et al. reported that lean-MAFLD had the lowest risk of CVD, followed by OW-MAFLD without MA, OW-MAFLD with MA, and DM-MAFLD (6). Collectively, it appears that the majority of young women with PCOS have OW-MAFLD without MA, and could be at a high risk for future CVD. Therefore, it is crucial to establish effective interventions, such as weight loss programs to transition from OW-MAFLD to lean-MAFLD subtype or to prevent progression to OW-MAFLD with MA or DM-MAFLD subtypes, which are at high risk for CVD.

We observed that women with PCOS and MAFLD had higher proportions of HA and OA than women with PCOS without MAFLD. Logistic regression analysis also showed that HA and OA, but not PCO, had a significant impact on the odds of developing MAFLD. Several studies have demonstrated that among the PCOS phenotypes, HA+OA+PCO and HA+OA are associated with metabolic abnormalities such as insulin resistance, metabolic syndrome, and CVD. O’Reily et al. reported that in PCOS adipose tissues, androgen production is increased along with an elevated expression of AKR1C3 (an androgen-activating enzyme). Increased androgen levels stimulate lipid accumulation and inhibit lipolysis, ultimately leading to adipocyte hypertrophy and insulin resistance (19). Jones et al. compared hepatic steatosis between women with PCOS and a control cohort matched for age and BMI. The study showed that PCOS women with HA have significantly higher liver fat content than PCOS women without HA and controls after adjusting for insulin resistance (20). Multiple epidemiological studies have reported that HA is independently associated with an increased risk of NAFLD in PCOS (21–24).

Notably, PCOS women with HA+PCO have a lower MAFLD prevalence than PCOS women with OA+PCO. This suggests that HA might not be a significant contributor to MAFLD prevalence in women with PCOS. However, similar results were not observed for NAFLD prevalence. In the PCOS phenotypes, NAFLD prevalence was 33.6% in the HA+OA+PCO phenotype, 36.7% in the HA+OA phenotype, and 35.4% in the HA+PCO phenotype. The prevalence of NAFLD in PCOS women with OA+PCO was the lowest, at 25.2%. Barber et al. reported that PCOS women with OA+PCO had lower insulin resistance than PCOS women with HA+OA or HA+PCO phenotypes, and similar levels of HDL cholesterol and triglycerides (25). Welt et al. demonstrated that among women with PCOS, the prevalence of metabolic syndrome was the highest in women with HA+OA phenotype, followed by women with HA+PCO and OA+PCO phenotypes (26). Nonetheless, when the data was stratified by age, this trend achieved statistically significance exclusively within the 30–39 years age group. A key difference between the previous studies and our study is that we investigated the association between PCOS and MAFLD, and not insulin resistance, metabolic syndrome, or NAFLD. Additionally, our study focused on young Asian women with PCOS with an average age of 25 years. Nevertheless, at least in terms of MAFLD risk, it might be hypothesized that OA in women with PCOS significantly affected the incidence of MAFLD compared with the HA. OA could be considered a functional abnormality of the reproductive phenotype in PCOS, whereas PCO could be represented as a structural abnormality of the reproductive phenotype in PCOS. Further biological and epidemiological studies with larger sample sizes are necessary to explore the distinct roles of each PCOS phenotype.

The strength of this study lies in the robust recruitment of a substantial cohort comprising 1,422 young women with PCOS, thereby ensuring a homogeneous study population. To the best of our knowledge, this study is the first to examine the prevalence and clinical differences between NAFLD and MAFLD. Young women with PCOS have a higher risk of metabolic diseases including NAFLD, MAFLD, and cardiovascular diseases compared with women without PCOS. As younger women with PCOS have a longer life expectancy than the older women, identifying factors that can predict disease occurrence in this group and establishing early prevention strategies is crucial.

This study had several limitations. First, this was a cross-sectional study. Therefore, we could not confirm the causal effect of PCOS phenotypes on MAFLD occurrence. Second, we defined NAFLD diagnosis using the HSI index instead of the gold standard methods of imaging or liver biopsy. However, studies that have validated the HSI index against the gold-standard methods have reported that the HSI index exhibited fair-to-good diagnostic ability in identifying NAFLD (14–16). Third, the definition of HA in PCOS was limited to total testosterone due to constraints in uniformly available data. Androgen excess in PCOS originate from both ovarian and adrenal sources and measuring additional androgens such as androstenedione, dihydrotestosterone, dehydroepiandrosterone, and 11-ketotestosterone could have improved diagnostic accuracy (27, 28). Forth, cases of lean-MAFLD were absent, and the number of DM-MAFLD cases was relatively small, consequently limiting the statistical analysis for these specific groups. Nevertheless, the overwhelming majority of the approximately 1,500 young women with PCOS and MAFLD within our cohort were obese. To gain a more comprehensive understanding and to stratify risk factors, further research with larger sample sizes is imperative, particularly in assessing the prevalence of lean-MAFLD and DM-MAFLD. Lastly, we measured testosterone levels using a chemiluminescent immunoassay, a method generally acknowledged for its relatively lower accuracy than techniques employing mass spectrometry.

In conclusion, our study involving young Korean women with PCOS revealed a noteworthy MAFLD prevalence of 20.3%, with the predominant subtype being OW-MAFLD. Among the PCOS phenotypes, the HA+OA+PCO and HA+OA phenotypes showed an independent association with MAFLD. Among PCOS diagnostic components, HA and OA were associated with MAFLD. These findings underscore the importance for clinicians to identify high-risk subgroups among young women with PCOS, consider early screening practices, and implement targeted interventions to mitigate the development and progression of MAFLD in this population.

## Data Availability

The original contributions presented in the study are included in the article/**Supplementary Material**. Further inquiries can be directed to the corresponding author.

## Supplementary material

Supplementary Figure 1 Study flow. PCOS, polycystic ovary syndrome; ESHRE, european society of human reproduction and embryology; EUMC, ewha womans university medical center; NAFLD, nonalcoholic fatty liver disease; MAFLD, metabolic dysfunction-associated fatty liver disease; OW, overweight; MA, metabolic abnormalities.
