# Supplementary figures and images for: The association between phenotypes of polycystic ovary syndrome and metabolic dysfunction-associated fatty liver disease
Source: Front Endocrinol (Lausanne). 2025 Aug 4;16:1480528. doi: 10.3389/fendo.2025.1480528 (PMC12358263; doi:10.3389/fendo.2025.1480528)

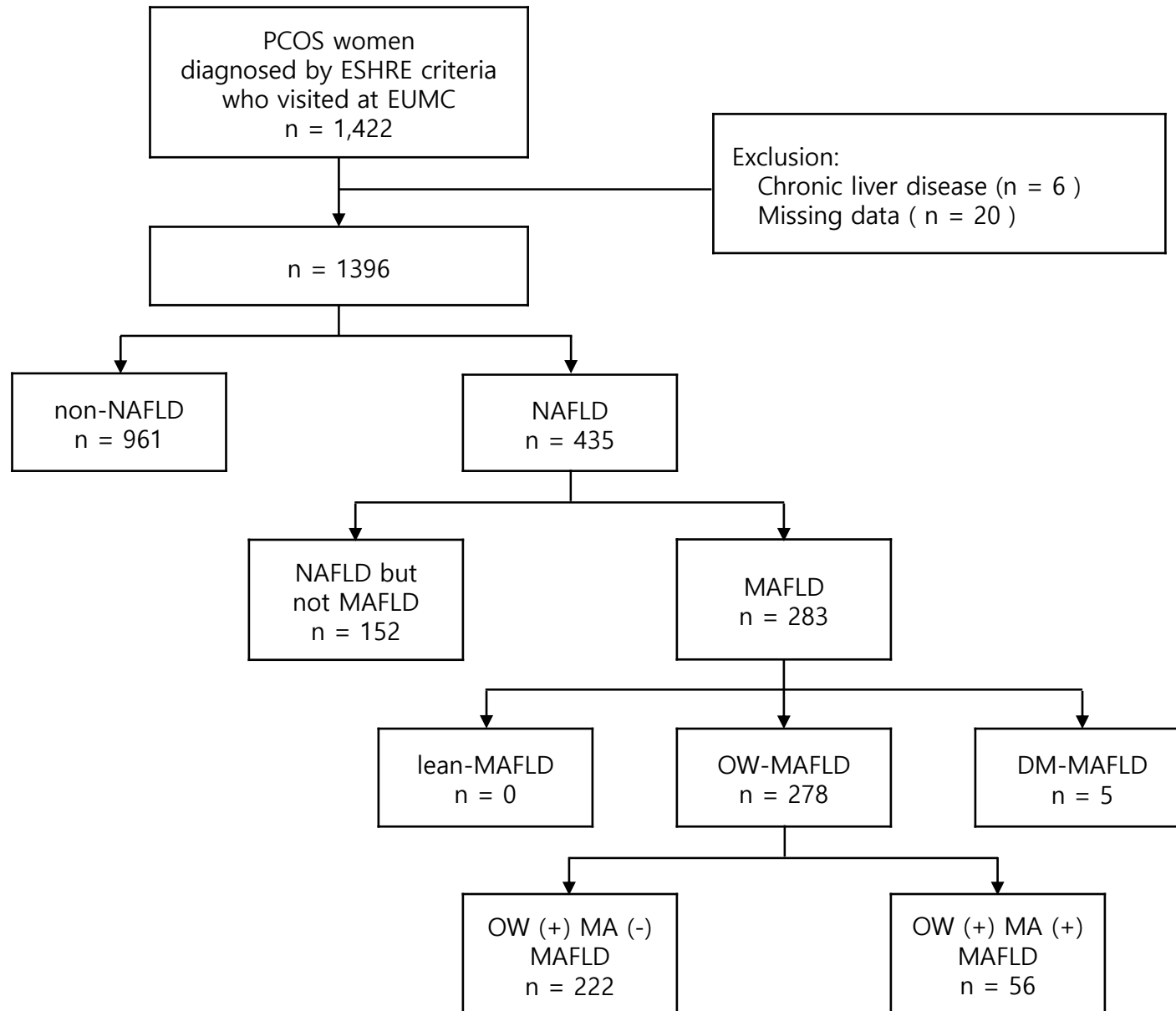

Supplement: Supplementary Figure 1 — Study flow. PCOS, polycystic ovary syndrome; ESHRE, european society of human reproduction and embryology; EUMC, ewha womans university medical center; NAFLD, nonalcoholic fatty liver disease; MAFLD, metabolic dysfunction-associated fatty liver disease; OW, overweight; MA, metabolic abnormalities. [file DataSheet1.pdf]
